# Impact of maternal cardiometabolic status after bariatric surgery on the association between telomere length and adiposity in offspring

**DOI:** 10.1038/s41598-023-47813-2

**Published:** 2023-11-26

**Authors:** Rodrigo San-Cristobal, Juan de Toro-Martín, Frédéric Guénard, Louis Pérusse, Simon Biron, Simon Marceau, Annie Lafortune Payette, Marie-Claude Vohl

**Affiliations:** 1 Centre Nutrition, santé et société (NUTRISS), Université Laval, Quebec, QC G1V 0A6 Canada; 2 Institut sur la nutrition et les aliments fonctionnels (INAF), Université Laval, Quebec, QC Canada; 3 School of Nutrition, Université Laval, Quebec, QC Canada; 4 Department of Kinesiology, Université Laval, Quebec, QC Canada; 5 Department of Surgery, Université Laval, Quebec, QC Canada; 6 Institut universitaire de cardiologie et de pneumologie de Québec, Université Laval, Quebec, Canada

**Keywords:** Endocrine system and metabolic diseases, DNA methylation

## Abstract

The impact of bariatric surgery on metabolic and inflammatory status are reflected in the epigenetic profile and telomere length mediated by the changes in the metabolic status of the patients. This study compared the telomere length of children born before versus after maternal bariatric surgery as a surrogate to test the influence of the mother’s metabolic status on children’s telomere length. DNA methylation telomere length (DNAmTL) was estimated from Methylation-EPIC BeadChip array data from a total of 24 children born before and after maternal bariatric surgery in the greater Quebec City area. DNAmTL was inversely associated with chronological age in children (r = − 0.80, p < 0.001) and significant differences were observed on age-adjusted DNAmTL between children born before versus after the maternal bariatric surgery. The associations found between body mass index and body fat percentage with DNAmTL in children born after the surgery were influenced by maternal triglycerides, TG/HDL-C ratio and TyG index. This study reports the impact of maternal bariatric surgery on offspring telomere length. The influence of maternal metabolic status on the association between telomere length and markers of adiposity in children suggests a putative modulating effect of bariatric surgery on the cardiometabolic risk in offspring.

## Introduction

Obesity is a complex chronic disorder involving many different factors such as education, eating habits and physical activity, as well as genetic and epigenetic factors and their interactions^1^. Among these interactions, sedentary behaviour and the consumption of energy-dense foods promote the development of obesity through the modulation of epigenetic factors in genes related to metabolic homeostasis and aging^2^. The rising rates of obesity worldwide, together with the improvement and decreasing costs of surgical procedures, is driving the increase in bariatric surgery for body weight management^3^. To date, bariatric surgery is the most effective treatment for severe obesity in the long-term and is associated with nutritional changes having a beneficial impact on the metabolic and inflammatory status of patients^4^.

The recent development of clinical epigenetics has accelerated the analysis of the relationship between cardiometabolic risk and epigenetic factors^5^. In this regard, recent studies have explored the association between the cardiometabolic health improvement observed following bariatric surgery and DNA methylation profiles in blood and target metabolic tissues. These results suggested that the blood epigenetic profile reflects, at least partially, the metabolic changes induced after bariatric surgery in individuals with severe obesity^6, 7^. In this regard, recent meta-analyses suggested that epigenetic modifications could explain the association between body weight loss and cardiometabolic benefits^8, 9^. In addition to the link between the improvements of the cardiometabolic profile and the differential methylation marks related with regulatory cell pathways, a recent study also reported a reduction in epigenetic age acceleration after bariatric surgery in patients with severe obesity^10^.

Telomere length is one of the most studied factors associated with cellular lifespan, aging and the development of age-related diseases^11^. Despite evidence linking telomere length reduction to increased adiposity in adults^12^ and children^13^, results regarding the effects of bariatric surgery on telomere shortening are still inconsistent^14^. Moreover, it has been suggested that perinatal factors playing a key role in offspring telomere length are also influenced by maternal epigenetics^15^. The increase in bariatric procedures in women of childbearing age is encouraging the study of the effects of these surgeries on developmental markers^16, 17^, but the impact on epigenetic factors in children born after the surgery remain unknown or poorly studied^18^. Nevertheless, recent studies reported an association between maternal overweight, but not paternal, with shorter telomeres in offspring, highlighting the importance of maternal weight management for a healthy aging of children^19, 20^.

In the present study, we compared an epigenetic estimate of telomere length of children born before versus after a maternal bariatric surgery, and we analyzed the influence of the metabolic status of the mother at the time of delivery on telomere shortening.

## Results

A total of 24 children, 12 born before and 12 born after their mother’s bariatric surgery, were eligible for the present study, after excluding individuals with missing information (Supplementary Fig. 1). Differences in anthropometric variables of mothers before and after the surgery are presented in Table 1. The age of the mothers was 27.1 years and 34.2 years at the delivery of the children before and after the bariatric surgery respectively. The time between the surgery and the birth of offspring (data not shown) varied from 0.6 to 11.0 years for the children born before and from 1.2 to 9.4 for those born after the surgery. Significant decreases in body weight (125.8 ± 4.5 to 80.6 ± 16.5 kg; p < 0.001) and body fatness indices (body mass index (BMI): 48.0 ± 7.7 to 31.0 ± 6.9 kg/m^2^, p < 0.001; waist circumference: 136.6 ± 13.5 to 100.9 ± 18.7 cm, p < 0.001; and waist-height ratio: 0.85 ± 0.09 to 0.63 ± 0.12, p = 0.001) were observed after the surgery. Body weight loss was accompanied by a significant improvement of the plasma lipid profile. Specifically, significant reductions in total-cholesterol (total-C; 4.6 ± 0.9 to 3.8 ± 0.6 mmol/l; p = 0.04), LDL-cholesterol (LDL-C; 2.6 ± 0.8 to 1.8 ± 0.5 mmol/l; p = 0.01), and triglyceride levels (TG; 1.8 ± 0.8 to 1.2 ± 0.5 mmol/l; p = 0.05) were observed, together with an increase in HDL-cholesterol levels (HDL-C; 1.1 ± 0.3 to 1.5 ± 0.5 mmol/l; p = 0.04). No significant reduction in plasma glucose levels was observed after the surgery (5.21 ± 1.61 to 4.60 ± 0.39 mmol/l; p = 0.25). Metabolic indices also exhibited a reduction of LDL-C/HDL-C ratio (2.51 ± 1.24 to 1.32 ± 0.68, p = 0.01), Non-HDL-C/HDL-C ratio (3.36 ± 1.72 to 1.74 ± 0.97 p = 0.011), TG/HDL-C ratio (1.78 ± 1.08 to 0.93 ± 0.67 p = 0.04), and triglyceride-glucose (TyG) index (8.82 ± 0.31 to 8.31 ± 0.39, p = 0.004).

**Table 1:** Characteristics of mothers before and after the bariatric surgery.

	Before	After	p
Clinical parameters			
Age at delivery (years)	27.1 (4.5)	34.2 (3.5)	**0.001**
Parity^a^	1 [1, 2]	2 [2–4]	** < 0.001**
Weight (kg)	125.8 (17.9)	80.6 (16.5)	** < 0.001**
BMI (kg/m^2^)	48.0 (7.7)	31.0 (6.9)	** < 0.001**
Waist circumference (cm)	136.6 (13.5)	100.9 (18.7)	** < 0.001**
Waist-height ratio	0.85 (0.09)	0.63 (0.12)	**0.001**
Fasting glucose (mmol/l)	5.21 (1.61)	4.60 (0.39)	0.25
Total-cholesterol (mmol/l)	4.56 (0.85)	3.83 (0.60)	**0.04**
HDL-cholesterol (mmol/l)	1.13 (0.25)	1.52 (0.51)	**0.04**
LDL-cholesterol (mmol/l)	2.58 (0.79)	1.76 (0.48)	**0.01**
Triglycerides (mmol/l)	1.82 (0.82)	1.18 (0.51)	**0.05**
Metabolic indices			
LDL-C/HDL-C ratio	2.51 (1.24)	1.32 (0.68)	**0.01**
Non-HDL-C/HDL-C ratio	3.36 (1.72)	1.74 (0.97)	**0.011**
TG/HDL-C ratio	1.78 (1.08)	0.93 (0.67)	**0.04**
TyG index	8.82 (0.31)	8.31 (0.39)	**0.004**

Variables are expressed as means (standard deviation) (n = 11). p stands for p-value for two-tailed paired t-test. Significant differences are in bold.

^a^Parity was expressed as median [range] and Mann–Whitney U test.

Significant differences were only observed for age (19.8 ± 7.1 vs 12.8 ± 7.1 years old; p = 0.03) in children born before versus after the maternal surgery (Table 2). No significant differences of body fatness indices were found between the offspring before versus after the maternal bariatric surgery.

**Table 2:** Characteristics of children born before versus after the maternal bariatric surgery.

	Before	After	p
At birth characteristics			
Sex (Male (%))	6 (50.0)	4 (33.3)	0.68
Weight (kg)	3.2 (0.9)	2.67 (0.5)	0.10
Height (cm)	49.7 (4.5)	47.7 (2.5)	0.20
At visit characteristics			
Age (Years)	19.8 (7.1)	12.8 (7.1)	**0.03**
BMI z-score	1.06 (1.38)	1.00 (1.03)	0.90
Waist-height ratio	0.54 (0.11)	0.57 (0.12)	0.50
Body Fat (%)	31.2 (14.8)	29.5 (8.8)	0.76

Variables are expressed as means (standard deviation) (n = 24, except for body fat measurement; n = 22). p stands for p-value for two-tailed unpaired t-test. Significant differences are in bold.

**Figure 1:**
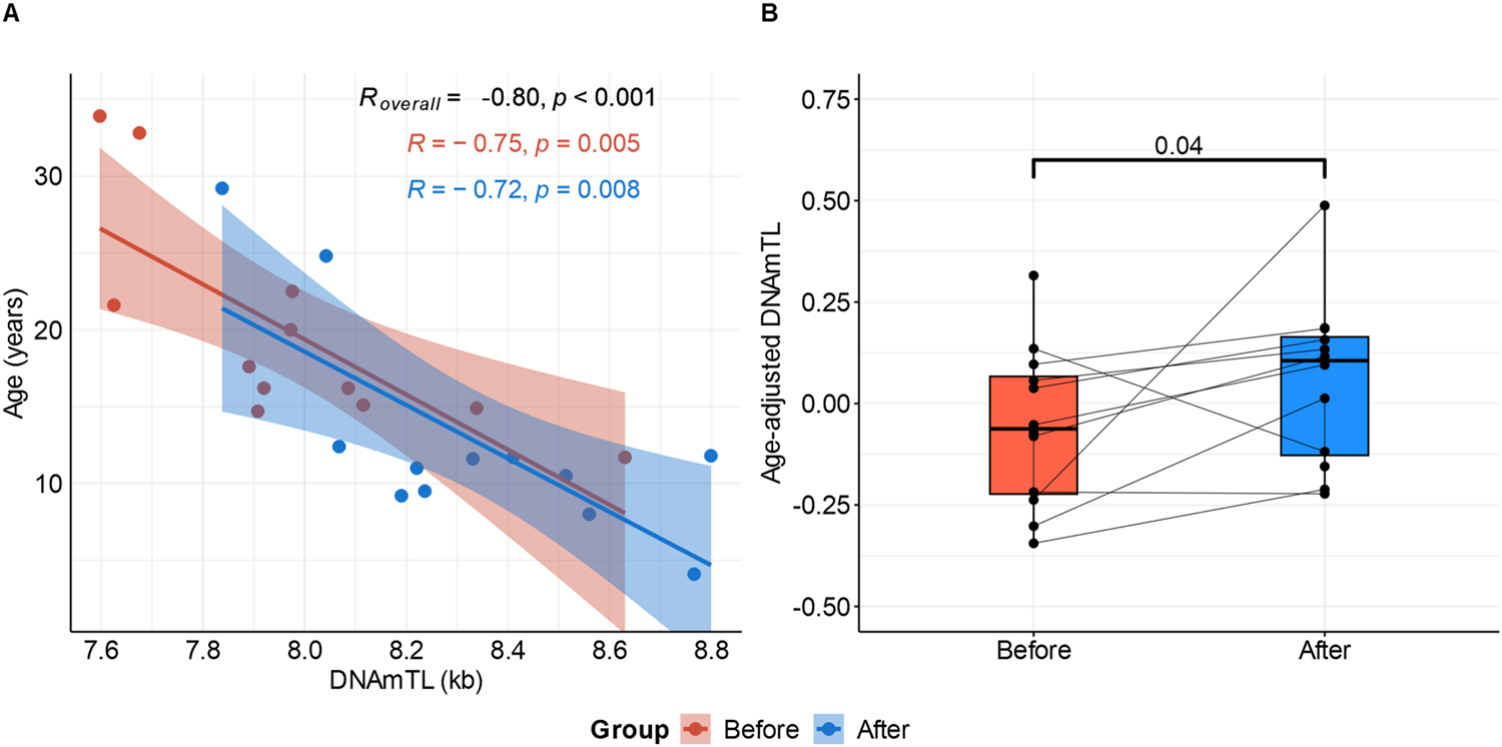
DNAmTL association with chronological age and differences in children born before versus after the maternal bariatric surgery. (**A**) Correlation between chronological age and DNAmTL by group. Pearson’s r and p-value for correlation between chronological age and DNAmTL are shown. (**B**) Differences of age-adjusted DNAmTL in blood samples of children born before versus after the maternal bariatric surgery.

In children, an inverse association between chronological age and telomere length, estimated from DNA methylation (DNAmTL), was observed in the overall group (r = − 0.80, p < 0.001), independently of the maternal bariatric surgery (Fig. 1A). Residuals for the regression of DNAmTL by chronological age were estimated to test for differences in telomere length between birth groups. Significant differences were found for the age-adjusted DNAmTL between children born before versus after the maternal bariatric surgery (Δ = 0.1, p = 0.04) (Fig. 1B).

**Figure 2:**
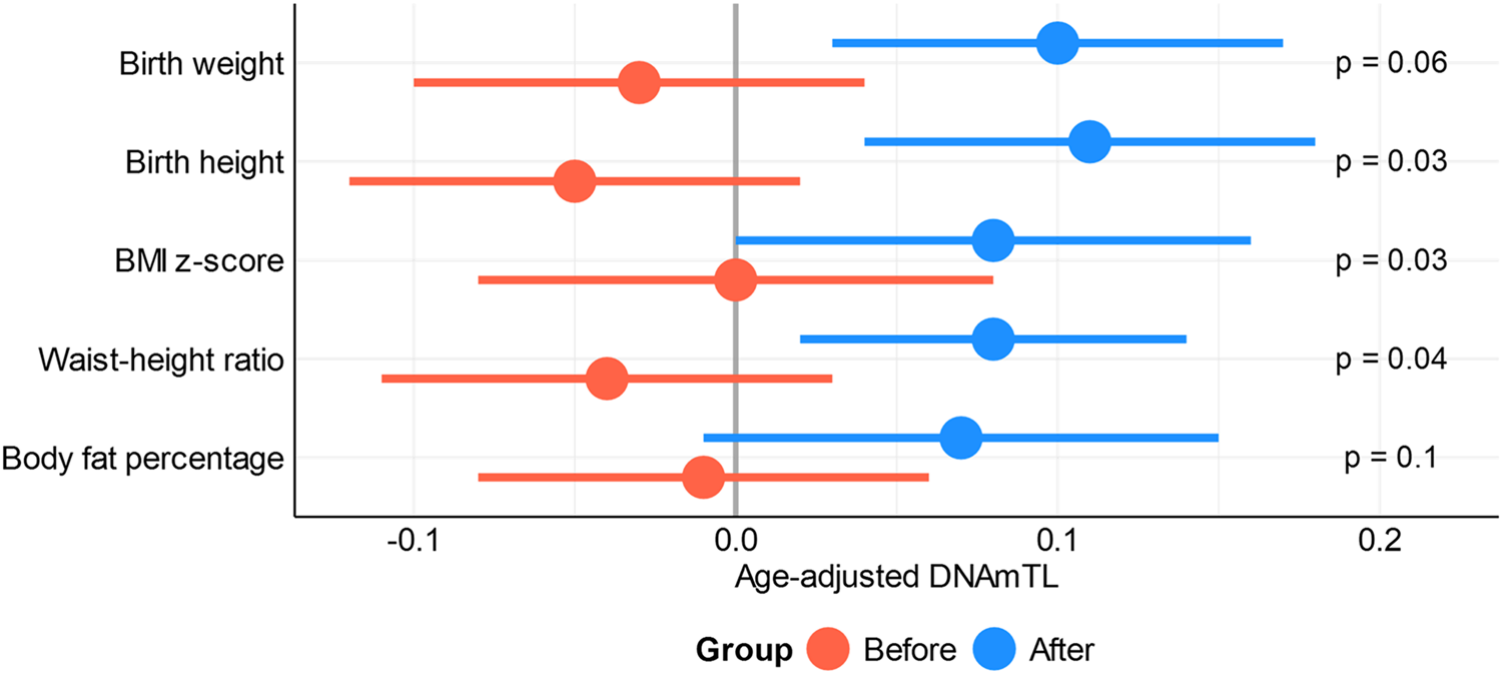
Differences in age-adjusted DNAmTL between children born before and after maternal bariatric surgery. The statistical models are adjusted for anthropometric measurements of children. p stands for p-values for contrast between children born before versus after the bariatric surgery.

Additionally, the interaction term between children’s characteristics and birth groups was added to the linear mixed model to further explore the effect of clinical parameters on DNAmTL differences (Fig. 2). Significant differences on DNAmTL were maintained after adjustment for birth height (p = 0.03), BMI z-score (p = 0.02) and waist-height ratio (p = 0.03). A trend was still observed for differences in birth weight (p = 0.06) and body fat percentage (p = 0.09) between birth groups.

**Figure 3:**
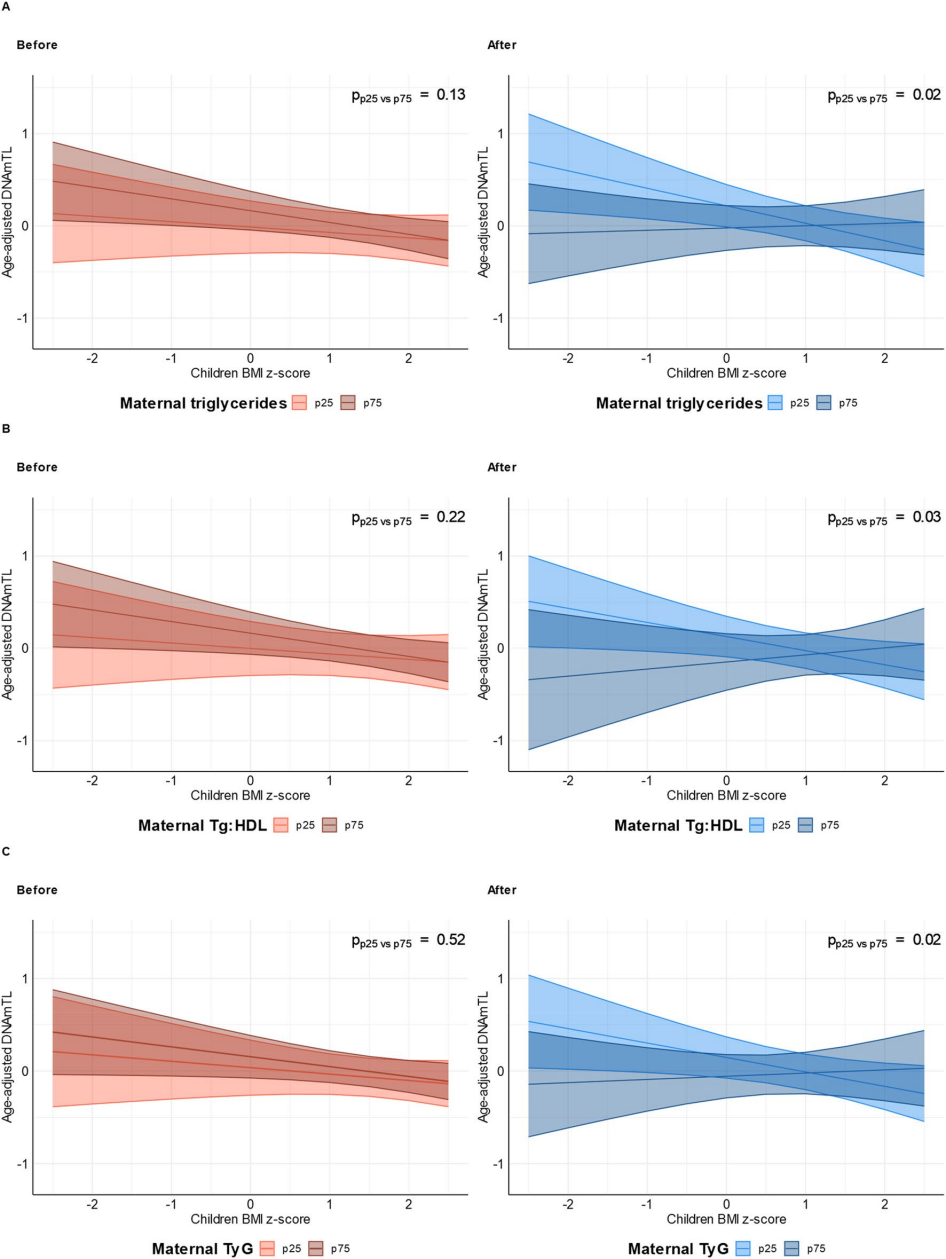
Modulatory effect of maternal metabolic status on the association between BMI z-score and age-adjusted DNAmTL in offspring. (**A**) Interaction between children’s BMI z-score and maternal triglycerides p = 0.008. Triglycerides percentiles estimated for birth group: Before: p25 = 1.30, p75 = 1.94; After: p25 = 0.85, p75 = 1.35. (**B**) Interaction between children’s BMI z-score and maternal TG/HDL-C ratio (Maternal TG:HDL-C) p = 0.026. TG/HDL-C percentiles estimated for birth group: Before: p25 = 0.92, p75 = 1.76; After: p25 = 0.49, p75 = 1.13. (**C**) Interaction between children’s BMI z-score and maternal TyG index (Maternal TyG) p = 0.009. TyG percentiles estimated for birth group: Before: p25 = 8.6, p75 = 8.9; After: p25 = 8.1, p75 = 8.5.

Finally, a complete interaction model including the interaction between birth group (before versus after the surgery), children’s adiposity markers and maternal metabolic status indices was built to analyze the impact of maternal metabolic status on DNAmTL of children (Supplementary Table 1). Maternal metabolic status indices showed significant interactions with BMI z-score and body fat percentage (Supplementary Table 1). More specifically, BMI z-score and body fat percentage showed similar change with significant interactions for group being born before versus after bariatric surgery (Fig. 3 and Supplementary Fig. 2) for triglycerides (BMI z-score: Δ = 0.21, p = 0.02; body fat percentage: Δ = 0.02, p = 0.13), TG/HDL-C ratio (BMI z-score: Δ = 0.23, p = 0.03; body fat percentage: Δ = 0.03, p = 0.06) and TyG index (BMI z-score: Δ = 0.19, p = 0.02; body fat percentage: Δ = 0.03, p = 0.03).

## Discussion

The present study reports the effect of maternal bariatric surgery on telomere length in the offspring. In addition, this study hypothesized about the potential role of the maternal metabolic improvements after the bariatric surgery on the relationship between telomere shortening and adiposity in the offspring. Present findings support the impact of the maternal plasma lipid profile and insulin resistance status (assessed by TyG index) on the association between age-adjusted telomere length and adiposity markers of children.

Bariatric surgery has been associated with beneficial effects on insulin sensitivity and on the plasma lipid profile^21^. More specifically, an improvement of the atherogenic-related lipoprotein profile, by reducing plasma LDL-C levels and LDL particle size, together with increasing HDL-C levels, has been observed following bariatric surgery^22^. These benefits seem to be associated with the degree of body weight reduction achieved^23^ and to its long-term maintaining after surgery^24^, therefore reducing the cardiometabolic risk of these patients. Accordingly, in the present study, bariatric surgery was associated in mothers with an improved cardiometabolic risk profile. A recent longitudinal study carried out in twins, reported on the association between changes in plasma lipid profile and methylation levels of *ABCG1*, *AKAP1* and *SREBF1*, three genes directly related with lipid metabolism^25^.

The development of new tools in the assessment of biological aging allowed to investigate the synchronisation between the chronological and the biological age^26^. Telomere shortening has been widely associated with aging and plays an important role in cellular function and senescence^27^. Herein, we observed that chronological age and telomere length was inversely correlated in both groups of children born before and after the bariatric surgery. In this respect, some authors have also suggested the significant impact of paternal age on TL^28^, which has been partially evaluated in the present study by including family features as random effects in the mixed model.

In addition to the inherent individual characteristics of age, sex and ethnicity affecting telomere length^29^, different modifiable factors, such as smoking habit, physical activity or drinking behaviour, has been proposed to accelerate telomere shortening^30^. These modifiable factors and their interactions with the genetic background are among those classically associated with an increased risk of developing cardiometabolic diseases^31^. Similarly, telomere length has been associated with a higher prevalence of cardiometabolic disorders, such as diabetes^32^, high blood pressure^33^ or cardiovascular events^34^. These findings may suggest that telomere length would play a major role on the reported association between modifiable risk factors and the development of cardiometabolic complications^35^. Indeed, the association between the increase of adiposity markers and telomere shortening found herein in children has been previously reported by several authors^32, 36^. All of this has led to the hypothesis that bariatric surgery would have a significant influence on telomere length. However, there are still some discrepancies about its actual impact^37, 38^. Thus, a prospective study with a 10-year follow-up after bariatric surgery suggested that there is an increase in telomere length induced by body weight loss^38^. Results of the present study support this hypothesis and suggest that the difference in telomere length found between children born before versus after the surgery may be also attributable to an imprinting effect of the metabolic improvements observed in mothers.

Maternal health risk factors and metabolic status during pregnancy have been proposed as determinants of epigenetic aging in the offspring, affecting both fetal growth and maturation, body size at birth and, ultimately, the future metabolic health of children^39^. This emphasizes the fact that changes in the environment during pregnancy would have an impact on the epigenetic age of the offspring^40^. Maternal circulating fatty acids, B-group vitamins, and homocysteine concentrations have been reported to be associated with an accelerated epigenetic gestational age of newborns^18, 41^. Furthermore, other authors have evidenced the key role of high n-3 polyunsaturated fatty acids in the maternal lipid profile during gestation with lower body fat mass and plasma TG levels, and higher HDL-C during childhood^42^. Consistent with these findings, our results suggest that maternal lipid markers may have an impact on telomere shortening in offspring, making children more sensitive to metabolic alterations during development. All these results pave the way for considering the use of epigenetic markers of biological age, more specifically epigenetic estimates of telomere length, as potential tool for assessing the risk of developing metabolic diseases. However, additional prospective analyses are still needed to strengthen the evidence and establish causality, in order to allow the design of decision aid tools for applications in precision medicine and precision nutrition^43^.

### Study strengths and limitations

Although the homogeneity of ethnicity in the present study sample is an advantage that allowed us to conduct a more robust analysis of the data, future studies in this area should include a greater diversity in order to generalize these results to other ethnicities. In addition, conducting studies in children was a challenge that limited the depth of the profiling on health indicators. In this regard, future studies should also include more accurate methods for estimating adiposity in children. The close collaboration with the medical team minimized these problems and reduced potential bias in recruitment and sampling for this study. Moreover, the assessment of nutritional status in children and young adults is an area where there is still considerable room of improvement^44^.

## Conclusion

These results suggest that there is an inherited effect of maternal bariatric surgery on offspring telomere length that may influence adiposity markers of children. In addition, this inherited effect of bariatric surgery on children’s metabolic health seems to be mediated by the maternal metabolic status.

## Methods

### Study population

A total of 24 children born to 11 French-Canadian women who had given birth before and after undergoing bariatric surgery in the Quebec City region were selected for the present study. The mothers had a biliopancreatic diversion with duodenal switch (BPD-DS) at the *Institut universitaire de cardiologie et de pneumologie de Québec* (IUCPQ) for the treatment of severe obesity (BMI > 30). Anthropometric measurements and blood sampling of mothers and children were performed at IUCPQ between August and October 2019. Adult women and the legal representative of minor children provided written informed consent for their participation. The study has been approved by the Ethics Committee of IUCPQ (2020-326421791).

### Sample collection and measurements

For mothers, data were collected during the post-surgery clinical follow-up using standardized procedures. For children, a visit was scheduled to the IUCPQ during which body weight, height, waist and hip circumference were measured. Body fat percentage was estimated using bioelectrical impedance analysis (TANITA Corp, Tokyo, Japan), and blood samples were taken from children for biochemical analysis.

Body mass index (BMI) for mothers was calculated dividing weight in kilograms by the square of height in meters. BMI z-score was estimated for children using charts for individuals between 2 and 20 years old, adapted for Canada from the World Health Organization^45^. In order to have a consistent metric of relative weight across offspring that could be analyzed as a continuous variable, reference values for BMI z-score at 20 years old were used for children older than 20 years old (n = 6), as suggested by Must and Anderson^46^. An additional waist-height ratio was estimated by dividing waist circumference by the height in centimeters for both mothers and children. Lipoprotein particle atherogenic ratios were estimated for mothers as follows: total-C to HDL-C (total-C/HDL-C ratio); non-HDL-C to HDL-C (non-HDL-C/HDL-C); TG to HDL-C (TG/HDL-C ratio). The TyG index was calculated using the following formula: TyG = Ln[TG (mg/dl) × fasting glucose (mg/dl)/2]^47^ to assess the maternal insulin resistance status^48^.

### Methylation DNA analysis and telomere length estimation

Blood samples from children were used to extract genomic DNA from isolated buffy coat using GenElute™ Blood Genomic DNA kit (Sigma, St Louis, MO, USA). Quality control and quantification of DNA were carried out by using NanoDrop spectrophotometer (Thermo Scientific, Wilmington, DE, USA) and PicoGreen DNA methods. Isolated DNA samples were bisulfite converted to perform the quantitative genome-wide methylation analysis conducted by using the Infinium Methylation-EPIC BeadChip array (Illumina, San Diego, CA, USA) according to manufacturer’s instructions at the Centre d’expertise et de services Génome Québec (Montreal, QC, Canada). Methylation reads were preprocessed and normalized using standard protocols with the *minfi* R package^49^. Probes mapped on sex chromosomes, overlapping with known single nucleotide polymorphisms or with cross reactive regions were removed. Data quality was assessed using multi-dimensional scaling to identify technical confounding bias and association with cell type composition. Beta values were estimated as the ratio of signal intensity of the methylated alleles to the sum of methylated and unmethylated intensity signals of the alleles. Telomere length was estimated from methylation data using the *methylclock* R package^26^, that includes the algorithm proposed by Lu et al. based on the methylation levels of 140 cytosine-phosphate-guanine dinucleotides for blood samples^50^.

### Statistical analysis

Differences in anthropometric and biochemical measurements before and after the bariatric surgery were analysed in mothers and children, using t-test and chi-square test, respectively for continuous and categorical variables. Pearson correlation coefficient between chronological age and DNAmTL was computed for children by birth group (before versus after the bariatric surgery). Due to the direct association showed between both variables, age-adjusted DNAmTL was estimated as residuals values from DNAmTL regressed by chronological age of children, this adjusted measurement was used to determine the effects of maternal surgery on the offspring removing the differences caused by aging. Linear mixed models with random effects by family (including mother id, same father and parity to avoid the effects of familiar relatedness between subjects) were performed using *lme4* and *emmeans* R packages^51^. An initial model adjusted for children’s sex was used to test the differences on age-adjusted DNAmTL between children born before and after the maternal surgery. An additional interaction term was included to estimate the effect of children’s adiposity markers on the variations of the age-adjusted DNAmTL. A subsequent model was constructed to evaluate the effects of the interaction between children’s adiposity markers and the metabolic status of mothers on age-adjusted DNAmTL of children. This model was adjusted for children’s sex, age of the mother at delivery and time in years from the surgery. A p-value ≤ 0.05 was considered as significant.

### Ethics approval and consent to participate

The study was approved according to the ethical standards of the Declaration of Helsinki by the Institut universitaire de cardiologie et de pneumologie de Québec ethic committee with the aprobal number: 2020-326421791. All participants provided written, informed consent.

### Supplementary Information

Supplementary Figure 1.

Supplementary Figure 2.

Supplementary Table 1.

## Data Availability

Datasets generated during and/or analyzed during the current study are available from the corresponding authors on reasonable request.
