# Supplementary figures and images for: Impact of maternal cardiometabolic status after bariatric surgery on the association between telomere length and adiposity in offspring
Source: Sci Rep. 2023 Nov 26;13:20771. doi: 10.1038/s41598-023-47813-2 (PMC10679094; doi:10.1038/s41598-023-47813-2)

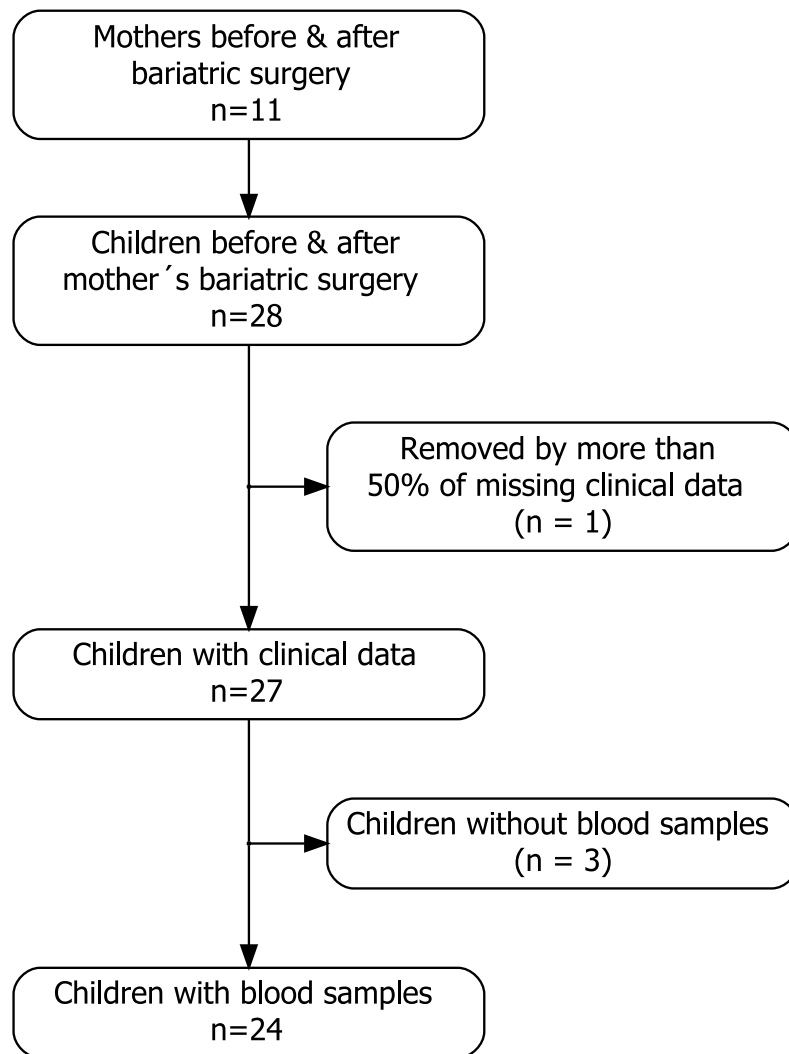

**Supplementary Figure 1:** Flowchart of study sample selection.

Supplement: Supplementary file 1 — Supplementary Figure 1. [file 41598_2023_47813_MOESM1_ESM.pdf]
