# Supplementary material for: Impact of maternal cardiometabolic status after bariatric surgery on the association between telomere length and adiposity in offspring
Source: Sci Rep. 2023 Nov 26;13:20771. doi: 10.1038/s41598-023-47813-2 (PMC10679094; doi:10.1038/s41598-023-47813-2)
Supplement: Supplementary file 2 — Supplementary Figure 2. [file 41598_2023_47813_MOESM2_ESM.pdf]

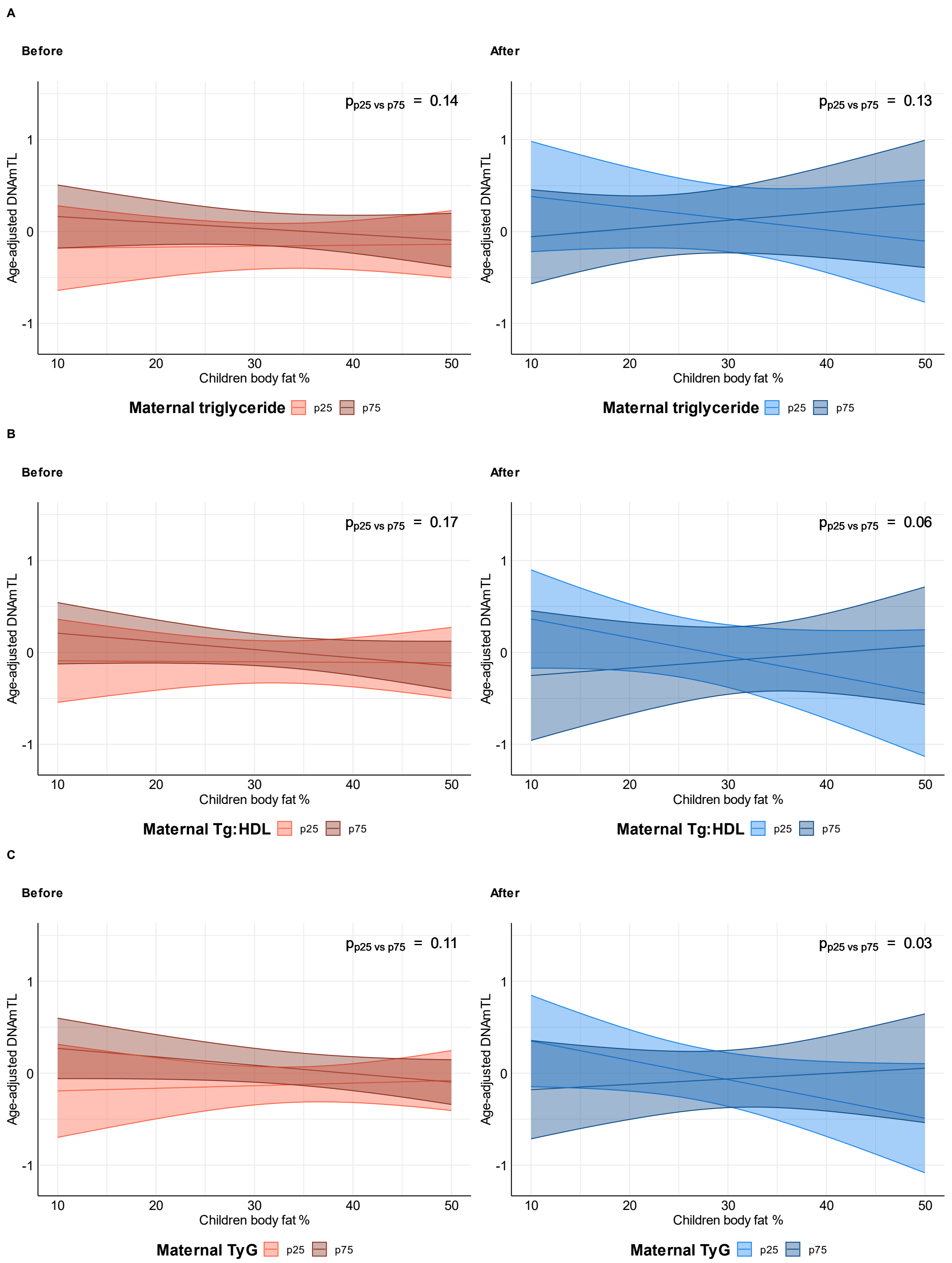

Supplementary Figure 2: Modulatory effect of maternal metabolic status on the association between percentage of body fat and age-adjusted DNAmTL in offspring. **A**: Interaction between children's body fat and maternal triglycerides p = 0.00813. Triglycerides percentiles estimated for birth group: Before: p25 = 1.30, p75 = 1.94; After: p25 = 0.85, p75 = 1.35. **B**: Interaction between children's body fat and maternal TG/HDL-C ratio (Maternal TG:HDL-C) p = 0.026. TG/HDL-C percentiles estimated for birth group: Before: p25 = 0.92, p75 = 1.76; After: p25 = 0.49, p75 = 1.13. **C**: Interaction between children's BMI z-score and maternal TyG index (Maternal TyG) p = 0.009. TyG percentiles estimated for birth group: Before: p25 = 8.6, p75 = 8.9; After: p25 = 8.1, p75 = 8.5.
