# Supplementary material for: Impact of maternal cardiometabolic status after bariatric surgery on the association between telomere length and adiposity in offspring
Source: Sci Rep. 2023 Nov 26;13:20771. doi: 10.1038/s41598-023-47813-2 (PMC10679094; doi:10.1038/s41598-023-47813-2)
Supplement: Supplementary file 3 — Supplementary Table 1. [file 41598_2023_47813_MOESM3_ESM.pdf]

Supplementary Table 1: Effect on age-adjusted DNAmTL of the interaction between anthropometric measurements of children and maternal metabolic indices.

	Before				After				p_{before}	p_{after}	p_{int}
Interaction with triglyceride											
	Percentil25 = 1.30		*Percentil75 = 1.94*		*Percentil25 = 0.85*		*Percentil75 = 1.35*				
	β	CI 95%	β	CI 95%	β	CI 95%	β	CI 95%			
Weight at birth	0.004	(-0.238 - 0.245)	0.125	(-0.208 - 0.458)	0.367	(-0.094 - 0.829)	0.085	(-0.195 - 0.366)	0.569	0.118	0.184
Height at birth	0.009	(-0.049 - 0.066)	0.022	(-0.074 - 0.119)	0.053	(-0.022 - 0.127)	0.028	(-0.062 - 0.117)	0.723	0.469	0.451
BMI z-score	-0.059	(-0.183 - 0.066)	-0.128	(-0.23 - -0.026)	-0.189	(-0.331 - -0.048)	0.025	(-0.132 - 0.182)	0.129	**0.018**	**0.008**
Waist-height ratio	-1.508	(-3.332 - 0.316)	-1.46	(-3.734 - 0.814)	-0.759	(-2.000 - 0.482)	0.715	(-2.102 - 3.532)	0.958	0.209	0.237
Body fat percentage	0.001	(-0.015 - 0.017)	-0.006	(-0.019 - 0.007)	-0.012	(-0.036 - 0.012)	0.009	(-0.015 - 0.033)	0.140	0.133	0.078
Interaction with TG/HDL-C ratio											
	Percentil25 = 0.92		*Percentil75 = 1.76*		*Percentil25 = 0.49*		*Percentil75 = 1.13*				
	β	CI 95%	β	CI 95%	β	CI 95%	β	CI 95%			
Weight at birth	0.036	(-0.228 - 0.299)	0.068	(-0.189 - 0.324)	0.291	(-0.179 - 0.762)	0.076	(-0.263 - 0.414)	0.843	0.221	0.261
Height at birth	0.007	(-0.045 - 0.059)	0.024	(-0.049 - 0.097)	0.042	(-0.030 - 0.115)	0.027	(-0.068 - 0.122)	0.572	0.637	0.522
BMI z-score	-0.059	(-0.200 - 0.082)	-0.126	(-0.236 - -0.016)	-0.153	(-0.291 - -0.015)	0.077	(-0.133 - 0.286)	0.215	**0.034**	**0.018**
Waist-height ratio	-1.701	(-3.626 - 0.224)	-1.399	(-3.471 - 0.674)	-0.701	(-1.910 - 0.509)	0.655	(-2.255 - 3.565)	0.682	0.217	0.316
Body fat percentage	-0.001	(-0.018 - 0.017)	-0.009	(-0.021 - 0.003)	-0.02	(-0.046 - 0.006)	0.008	(-0.019 - 0.036)	0.171	0.060	**0.031**
Interaction with TyG index											
	Percentil25 = 8.57		*Percentil75 = 8.90*		*Percentil25 = 8.09*		*Percentil75 = 8.49*				
	β	CI 95%	β	CI 95%	β	CI 95%	β	CI 95%			
Weight at birth	0.032	(-0.184 - 0.248)	0.065	(-0.192 - 0.322)	0.255	(-0.177 - 0.686)	0.033	(-0.301 - 0.366)	0.801	0.218	0.288
Height at birth	0.010	(-0.035 - 0.055)	0.019	(-0.062 - 0.100)	0.042	(-0.023 - 0.107)	0.028	(-0.073 - 0.130)	0.765	0.657	0.630
BMI z-score	-0.065	(-0.212 - 0.081)	-0.106	(-0.213 – 0.000)	-0.160	(-0.301 - -0.02)	0.030	(-0.143 - 0.202)	0.522	**0.024**	**0.041**
Waist-height ratio	-1.579	(-3.520 - 0.361)	-0.869	(-3.205 - 1.466)	-0.758	(-1.922 - 0.406)	0.158	(-2.168 - 2.485)	0.501	0.287	0.97
Body fat percentage	0.004	(-0.016 - 0.024)	-0.009	(-0.020 - 0.002)	-0.022	(-0.045 - 0.002)	0.005	(-0.017 - 0.028)	0.112	**0.028**	**0.011**
